# Prednisolone as Preservation Additive Prevents from Ischemia Reperfusion Injury in a Rat Model of Orthotopic Lung Transplantation

**DOI:** 10.1371/journal.pone.0073298

**Published:** 2013-08-29

**Authors:** Patrick Paulus, Johannes Holfeld, Anja Urbschat, Haitham Mutlak, Pia Alexandra Ockelmann, Sabine Tacke, Kai Zacharowski, Christin Reissig, David Stay, Bertram Scheller

**Affiliations:** 1 Clinic of Anesthesiology, Intensive Care Medicine and Pain Therapy, Goethe-University Hospital Frankfurt, Frankfurt am Main, Germany; 2 Clinic of Cardiac Surgery, Innsbruck Medical University, Innsbruck, Austria; 3 Goethe-University Hospital, Frankfurt am Main, Germany; 4 Department of Veterinary Clinical Sciences, Clinic for Small Animals Surgery, Justus-Liebig University, Giessen, Germany; University Hospital Münster, Germany

## Abstract

The lung is, more than other solid organs, susceptible for ischemia reperfusion injury after orthotopic transplantation. Corticosteroids are known to potently suppress pro-inflammatory processes when given in the post-operative setting or during rejection episodes. Whereas their use has been approved for these clinical indications, there is no study investigating its potential as a preservation additive in preventing vascular damage already in the phase of ischemia. To investigate these effects we performed orthotopic lung transplantations (LTX) in the rat. Prednisolone was either added to the perfusion solution for lung preservation or omitted and rats were followed for 48 hours after LTX. Prednisolone preconditioning significantly increased survival and diminished reperfusion edema. Hypoxia induced vasoactive cytokines such as VEGF were reduced. Markers of leukocyte invasiveness like matrix metalloprotease (MMP)-2, or common pro-inflammatory molecules like the CXCR4 receptor or the chemokine (C-C motif) ligand (CCL)-2 were downregulated by prednisolone. Neutrophil recruitment to the grafts was only increased in Perfadex treated lungs. Together with this, prednisolone treated animals displayed significantly reduced lung protein levels of neutrophil chemoattractants like CINC-1, CINC-2α/β and LIX and upregulated tissue inhibitor of matrix metalloproteinase (TIMP)-1. Interestingly, lung macrophage invasion was increased in both, Perfadex and prednisolone treated grafts, as measured by MMP-12 or RM4. Markers of anti-inflammatory macrophage transdifferentiation like MRC-1, IL-13, IL-4 and CD163, significantly correlated with prednisolone treatment. These observations lead to the conclusion that prednisolone as an additive to the perfusion solution protects from hypoxia triggered danger signals already in the phase of ischemia and thus reduces graft edema in the phase of reperfusion. Additionally, prednisolone preconditioning might also lead to macrophage polarization as a beneficial long-term effect.

## INTRODUCTION

Ischemia-reperfusion (IR) is mainly characterized by an overshooting inflammatory response leading to tissue edema [1–3] and probably resulting in primary graft dysfunction [4]. It affects an estimated 10 to 25% of all lung transplants [5]. Ischemia reperfusion injury is known to prime transplanted organs to be more susceptible for later rejection episodes [6–8]. Preventing the occurrence of such reperfusion damages might be an important therapeutic strategy in conserving the organ’s long-term function [5].

Although there are many therapeutic strategies for the treatment of acute complications, there is no direct causal therapy to prevent IR damage [5]. In a previous work, we could show that a direct inhibition of the hypoxia driven signaling pathways might be a beneficial approach to induce ischemia tolerance in organs and to improve outcome after transplantation. In that case we used Deguelin, a substance of poor solubility and poor tolerability at high doses, to inhibit hypoxia inducible factor (HIF) mediated inflammation. The main mode of action of Deguelin consists in the prevention of pulmonary edema formation by indirectly inhibiting the vascular endothelial growth factor (VEGF), a substance that is 50.000 times more potent in its pro-edematous action than histamine [4,9]. In our search for a better substance, prednisolone came into our focus. Glucocorticoids are well-characterized and well tolerable substances that play an important role in transplantation medicine.

Glucocorticoids have a broad mode of action by interfering with pro inflammatory gene expression mainly via nuclear factor (NF) κB Inhibition [10,11]. This mechanism leads to the suppression or modulation of many pro-inflammatory pathways and results in the inhibition of the recruitment of inflammatory cells into the newly transplanted organ [12]. Prednisolone mainly inhibits the loco-regional production of substances important for airway inflammation, namely interferon (IFN)-γ, CXCR4 and interleukin (IL)-6 [13–15]. These proteins are largely responsible for early graft inflammation following ischemia and reperfusion, especially after transplantation. Prednisolone acts anti-inflammatory by abrogating their expression already at a transcriptional level. However, glucocorticoids not only downregulate pro-inflammatory genes, but also enhance the production of anti-inflammatory proteins such as IL-4, IL-13 or IL-10 [16,17].

Reperfusion injury is mainly mediated through cellular inflammation which is a biphasic process involving a first line of attack by macrophages and later on by neutrophils [18–20]. Macrophages are able to quickly respond to pro-inflammatory stimuli and chemoattractants produced in alveolar cells during ischemia. The attractants induce a first line response in resident macrophages, which then potentiate inflammation by triggering the recruitment of other immune cells like neutrophils [21–24]. Whereas the classical function of macrophages is the unspecific removal of substances invading the body, there is increasing evidence that macrophages also modulate the inflammatory response. For several years now, the existence of anti-inflammatory or M2 macrophages has been described. More interestingly, with the increasing understanding of the biological functions and interplays of the different cyto- and chemokines, the underlying differentiation pathways from M1 to M2 macrophages could be elucidated [25]. Several gene products that play an important role in M2 differentiation have been described, such as macrophage mannose receptor (MRC)-1, IL-13, IL1 receptor antagonist (IL1ra) and CD163 [26–28]. For some diseases glucocorticoids have been observed to favor M2 polarization by interfering with the latter pathways [29]. In this complex pathway, IL-4, IL-10 and IL13 occupy a pivotal role. Glucocorticoids induce the production of IL-4 and IL-13, which in turn activate STAT-6, a signaling pathway member that finally induces M2 polarization by upregulating MRC-1 and CD163. IL-10 plays a rather differential role, some investigations describe IL-10 inducing macrophage polarization by activating STAT-3, which in turn synergizes with STAT-6 and results in MRC-1 and CD-163 upregulation [30–32]. IL-10 has also been described to play a context specific pro-inflammatory role that is regulated by spacio-temporal factors [4,33,34]. Regulation of these pathways is ensured by activation of SOCS-1 and -3, two signaling molecules that inhibit either STAT-6 or STAT-3 directly [35–37].

Other important cellular populations that play a crucial role in the pathogenesis of ischemia and reperfusion injury are neutrophils. Cytokine-induced neutrophil chemoattractant (CINC)-1, CINC-2α/β and lipopolysaccharide-induced CXC chemokine (LIX) are potent chemoattractants for this cell type. Their secretion is stimulated upon ischemia by resident cells like endothelial cells. Matrix-metalloproteinase (MMP)-2, which is inhibited by the tissue inhibitor of matrix metalloproteinase (TIMP)-1, has been shown to be induced by the same mechanisms and to activate these neutrophil specific chemoattractants [38]. MMP-2 is needed by the neutrophils to degrade the extracellular matrix, a process that is crucial for diapedesis. MMP-2 thus correlates with the invasiveness of these cells during inflammation [39]. Corticoids are known to be potent inhibitors of MMPs and thus prevent tissue inflammation and more specifically remodeling, as is the case in lung fibrosis [40,41], whereas their action on CINC-1, CINC-2α/β and LIX is largely unknown.

Usually, corticoids are administered in transplantation medicine before antigen contact is restored to induce immunotolerance immediately before restoration of the blood flow to the transplanted organ. However the strategy to use prednisolone as an additive to perfusion solutions might have been adopted by some transplantation centers. To our knowledge there are currently no experimental studies systematically investigating the influence of prednisolone on ischemia-induced inflammation.

We show that prednisolone not only suppresses reperfusion damage by reducing hypoxia induced signaling, namely vascular endothelial growth factor (VEGF)-A induced lung edema but also induces transdifferentiation of macrophages towards an anti-inflammatory phenotype, which in turn may play a significant role in long-term lung protection.

## Results

### Prednisolone treatment significantly increases short-term survival in rats following orthotopic lung transplantation (LTX)

**Figure 1:**
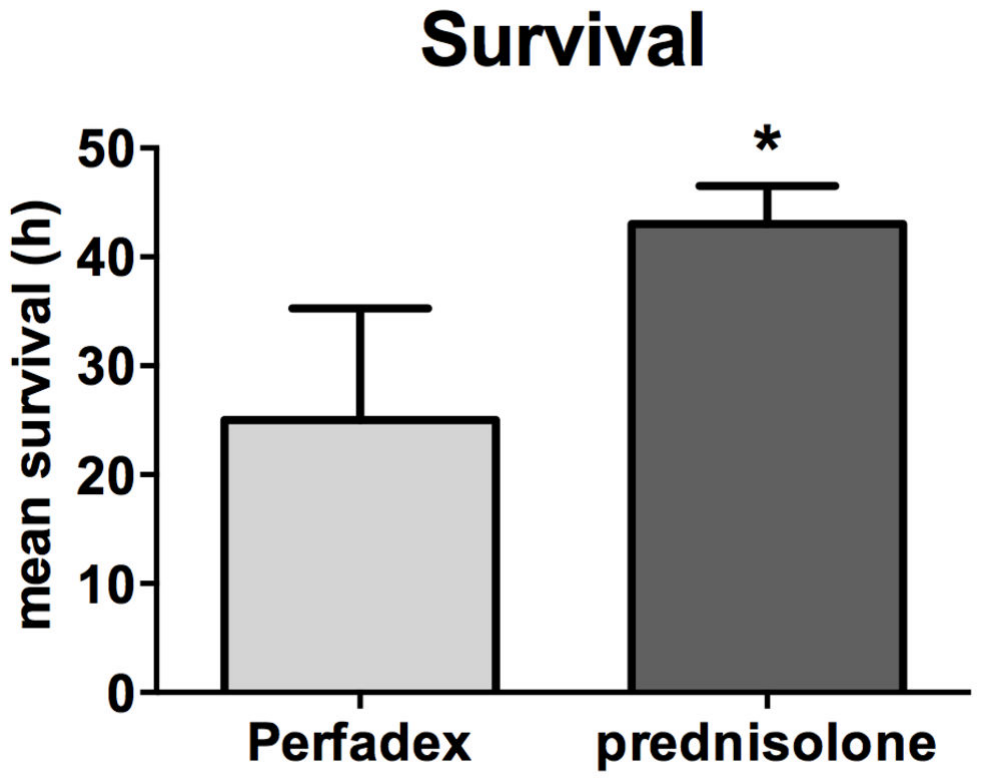
Prednisolone treatment significantly increases short-term survival following orthotopic lung transplantation (LTX). Mean short-term survival in hours following LTX. Survival within the observation period of 48h following LTX was tracked. Values are expressed as means ± standard error of the mean (SEM); significance level was set to P<0.05,:*, significantly different from Perfadex-group. Transplantation procedure was repeated until n=6 survivors per group were obtained to ensure homogenic comparison between the groups. ANOVA followed by Bonferroni’s post-hoc multiple comparisons test.

After LTX, animals were observed for 48 hours. Rats that were transplanted with Perfadex-treated lungs had significantly poorer short-term survival than the corresponding rats receiving prednisolone pre-conditioned lungs. Mean short-term survival was 25.00 ± 10.29 hours for Perfadex only treated lungs vs. 43.00 ± 3.52 hours for prednisolone-preconditioned lungs (p<0.05, Figure 1).

### Prednisolone preserves the macro- and microstructural aspect of the grafts and reduces reperfusion edema formation following LTX

**Figure 2:**
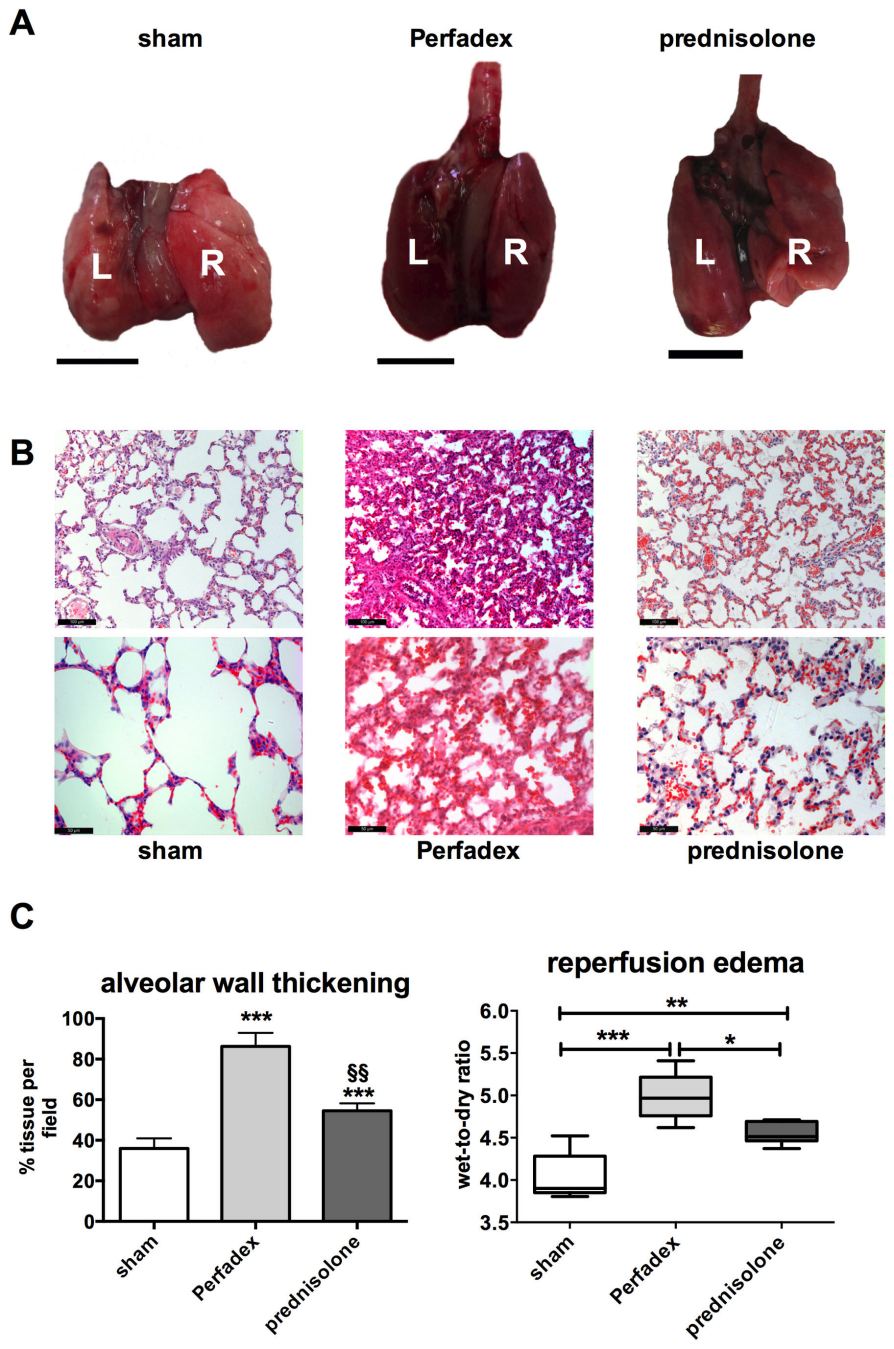
Prednisolone preserves the macro- and microstructural aspect of the grafts and reduces reperfusion edema formation following LTX. (**A**) Image shows explanted lungs 48 hours after LTX. Lungs from prednisolone-treated animals (right picture), Perfadex-treated lungs (middle picture) and sham lungs (left picture) were compared. L = left lobe, R = right lobe. (**B**) Representative micrographs (H&E staining) showing microstructural alterations and alveolar thickening as edema surrogate by directly comparing shams (left micrographs) with Perfadex-treated animals (middle) and prednisolone-preconditioned lungs (right pictures). (**C**) Quantification of the alveolar thickening by measuring the percentage of tissue occupying every slide (ratio tissue vs. no tissue, left graph). Values are expressed as % tissue/slide (n=3 random slides per animal). Quantification of the amount of edema (lung water) by evaluating the wet-to-dry ratio (right graph), n=6 per group. Calibration bar for macroscopic pictures (**A**) represents 1 cm. Calibration bar for H&E micrographs (**B**) represents 100 µm (200 x magnification, upper micrograph) and 50 µm (400 x magnification, lower micrograph). Significance level was set to P<0.05. P<0.05: *; P<0.01: **, § § and P<0.001: ***. * = significantly different from shams and § = significantly different from Perfadex-group. ANOVA followed by Bonferroni’s post-hoc multiple comparisons test.

The explanted lungs from the prednisolone group showed a better general macroscopic appearance. Lungs treated with Perfadex only had a dark colored aspect, indicating a higher fluid content. Prednisolone preconditioned lungs were almost identical in their macroscopic aspect to lungs from sham animals (Figure 2, A). Moreover, prednisolone preconditioned lungs had a preserved alveolar appearance and less perivascular or cellular edema 48h after reperfusion compared to the controls (Figure 2, B). For quantification of the edematous changes, the alveolar wall thickening was quantified using an automatized algorithm. Perfadex treated lungs had significantly larger alveoli whereas prednisolone-treated lungs were almost identical to sham lungs. By determining the amount of tissue per picture as described in the materials and methods section, the cellular swelling can be calculated. In Perfadex-treated lungs, 88.26 2.54% of the slide were covered by alveolar tissue, whereas in prednisolone-treated lungs only 54.56 2.54% of the slide consisted of lung tissue (P<0.001*, P<0.01§) (Figure 2, C left graph). For direct evaluation of the lung water as surrogate for edema, the wet-to-dry ratio was calculated. Again, Perfadex-treated 4.991 ± 0.10 (relative values) lungs had significantly increased tissue water than the prednisolone 4.549 ± 0.05 (relative values) preconditioned (P<0.05) and sham 4.034 ± 0.12 (relative values) lungs (P<0.001). Although prednisolone pretreated lungs had less edema than Perfadex treated lungs, their water content was significantly increased when compared to sham animals (P<0.01; Figure 2, C right graph).

### Prednisolone reduces hypoxia induced signaling and attenuates VEGF-A expression

**Figure 3:**
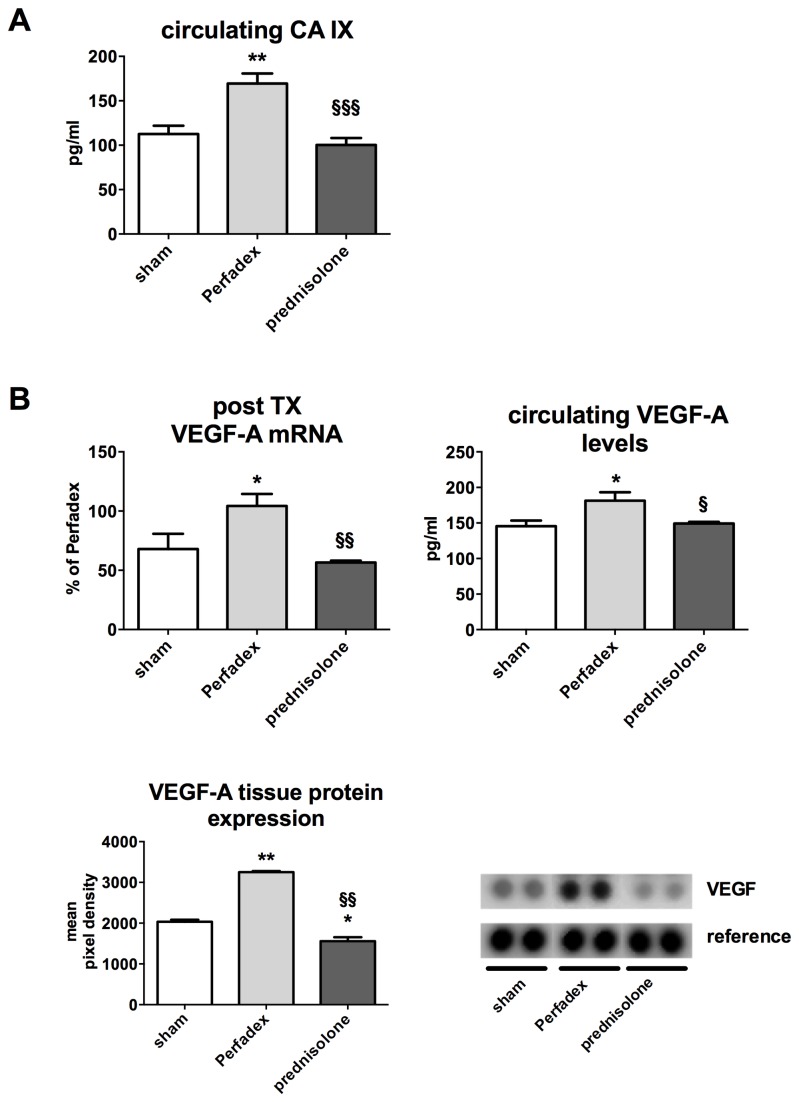
Prednisolone reduces hypoxia induced signaling and attenuates VEGF-A expression. . (**A**) Circulating carbonic anhydrase (CA) IX has been measured by ELISA in serum samples of all animals (values are expressed in pg/ml). (**B**) VEGF-A expression (mRNA, circulating and tissue protein) have been detected by RT-PCR (upper left graph, values expressed as % of Perfadex groups, Perfadex group has been set at 100%), ELISA (upper right graph, expression in pg/ml) and protein array (lower left graph, values expressed in mean pixel densities). Significance level was set to P<0.05. P<0.05: *, § and P<0.01: **, § § and P<0.001: § § §. * = significantly different from shams and § = significantly different from Perfadex-group. RT-PCR: experiments were performed in triplicate, protein array: pooled analysis in duplicate. ANOVA followed by Bonferroni’s post-hoc multiple comparisons test.

Serum concentrations of circulating carbonic anhydrase (CA) IX and VEGF-A were determined as surrogates for hypoxia induced signaling and reperfusion edema [4]. Circulating CA IX was significantly lower in animals that received prednisolone 100.1 ± 7.9 pg/ml when compared to controls with no treatment 169.5 ± 11.2 pg/ml (p<0.001). CA IX levels in the prednisolone group were almost comparable to the baseline levels (sham) 112.6 ± 9.3 (n.s.) (Figure 3, A). This observation correlated also with the wet-to-dry ratios (Figure 2 C). As expression of HIF-1 activation upon ischemia, post-operative VEGF-A expression directly correlated with reperfusion edema. VEGF-A mRNA was significantly blunted in prednisolone-treated animals 56.5 ± 1.7 and shams 67.9 ± 12.8 (expressed as % of the expression of the Perfadex group) compared to controls 104.3 ± 10.2% (p<0.05). Tissue VEGF-A protein expression was significantly upregulated in Perfadex-treated animals (3251 ± 50.32 mean pixel density) vs. sham (2034 ± 50.32 mean pixel density; P<0.01). Prednisolone treatment could significantly lower these expression levels even below normal values (1559 96.76 mean pixel density; P<0.05). Circulating VEGF-A detected in serum samples showed a significant upregulation of VEGF-A in controls 181.4 ± 11.8 pg/ml vs. shams 145.6 ± 7.9 pg/ml as well as in animals that received prednisolone 149.2 ± 2.3 pg/ml (p<0.05, Figure 3 B).

### Neutrophil recruitment is increased in lungs treated with Perfadex only and prednisolone treatment decreases neutrophil invasiveness

**Figure 4:**
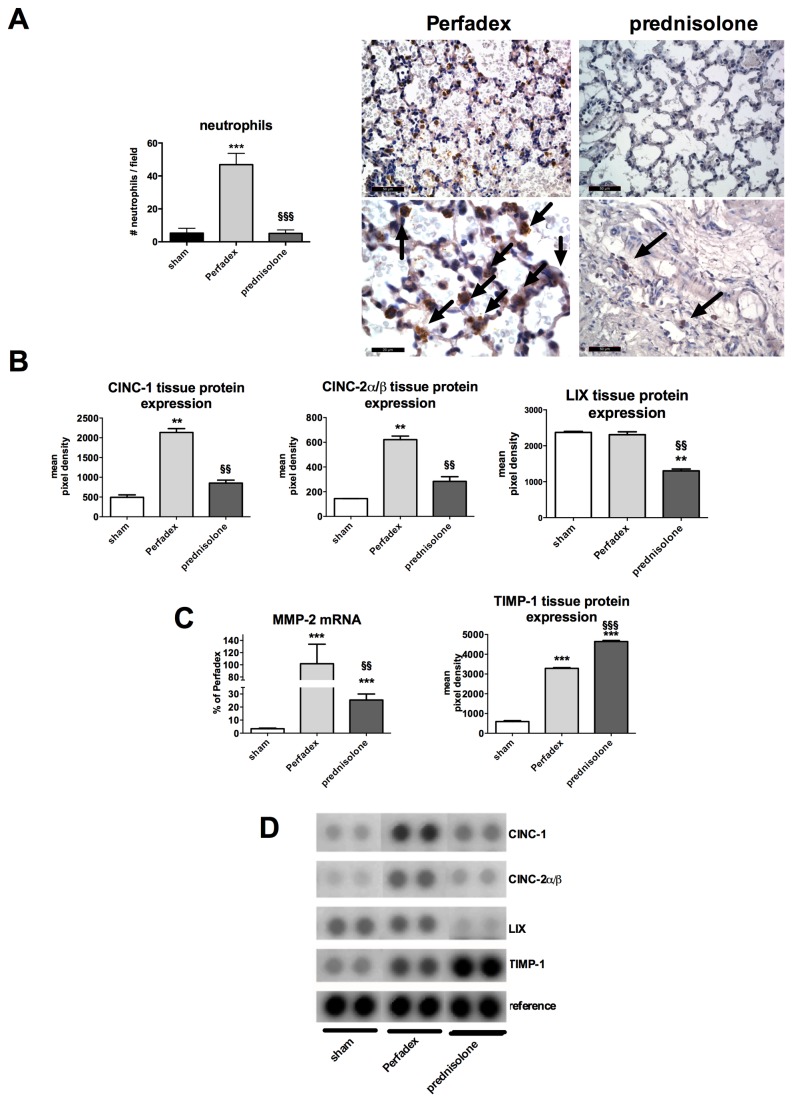
Prednisolone inhibits neutrophil invasion after LTX. (**A**) Specific neutrophil staining (arrows, micrographs) and quantification (left graph). Data are expressed as number of neutrophils/field. (**B**) Measurement of tissue protein expression of neutrophil chemoattractants in transplanted lungs by protein array. Data are expressed in mean pixel densities. (**C**) Evaluation of MMP-2 mRNA expression (measured by RT-PCR, data are expressed as % of Perfadex group, Perfadex group has been set at 100%) and its Inhibitor TIMP-1 by protein analysis (protein array). (**D**) Representative protein array pictures. Calibration bar for micrographs (**A**) represents 100 µm (200 x magnification, upper micrographs) and 50 µm (400 x magnification, lower micrographs). Significance level was set to P<0.05. P<0.01: **, § § and P<0.001: § § §. * = significantly different from shams and § = significantly different from Perfadex-group. RT-PCR: experiments were performed in triplicate, protein array: pooled analysis in duplicate. ANOVA followed by Bonferroni’s post-hoc multiple comparisons test.

Rats receiving lungs that had been treated with Perfadex only had significantly more neutrophil cells 46.88 ± 2.47 compared to prednisolone treated 5.12 ± 0.74 or sham lung cells 5.25 ± 1.04 (P<0.001, Figure 4 A). Prednisolone protects from hypoxia-induced inflammation by downregulating tissue expression of specific neutrophil chemoattractants such as CINC-1 (854.0 ± 74.43 mean pixel density), CINC-2α/β (283.5 ± 38.40 mean pixel density) and LIX (1300 ± 54.64 mean pixel density) vs. Perfadex (2134 ± 99.54, P<0.01; 621.5 ± 28.82, P<0.01 resp. 2309 ± 80.91, P<0.01 mean pixel density). Regarding LIX expression, prednisolone (1300 ± 54.64 mean pixel density) treatment resulted in lower levels compared to sham (2379 ± 28.8 mean pixel density, P<0.01) animals (Figure 4 B). Neutrophil invasiveness, which was quantified by MMP-2, was significantly lower in prednisolone-pretreated lungs than in Perfadex (101.9 ± 31.92%) treated ones (P<0.01). However, levels in prednisolone-treated lungs (25.38 ± 4.61%) were still significantly higher than in sham animals (3.37 ± 0.50%; P<0.001, Figure 4C). In contrast, the natural inhibitor of MMP-2, TIMP-1, was significantly upregulated in prednisolone-pretreated (4642 ± 57.36, mean pixel density) lungs compared to Perfadex (3285 ± 37.44, mean pixel density) treated lungs, whereas both interventional groups had significantly higher levels compared to sham (593.4 ± 46.56, mean pixel density) animals (P<0.001, Figure 4C).

### Prednisolone influences the lung macrophage composition in the reperfusion phase

**Figure 5:**
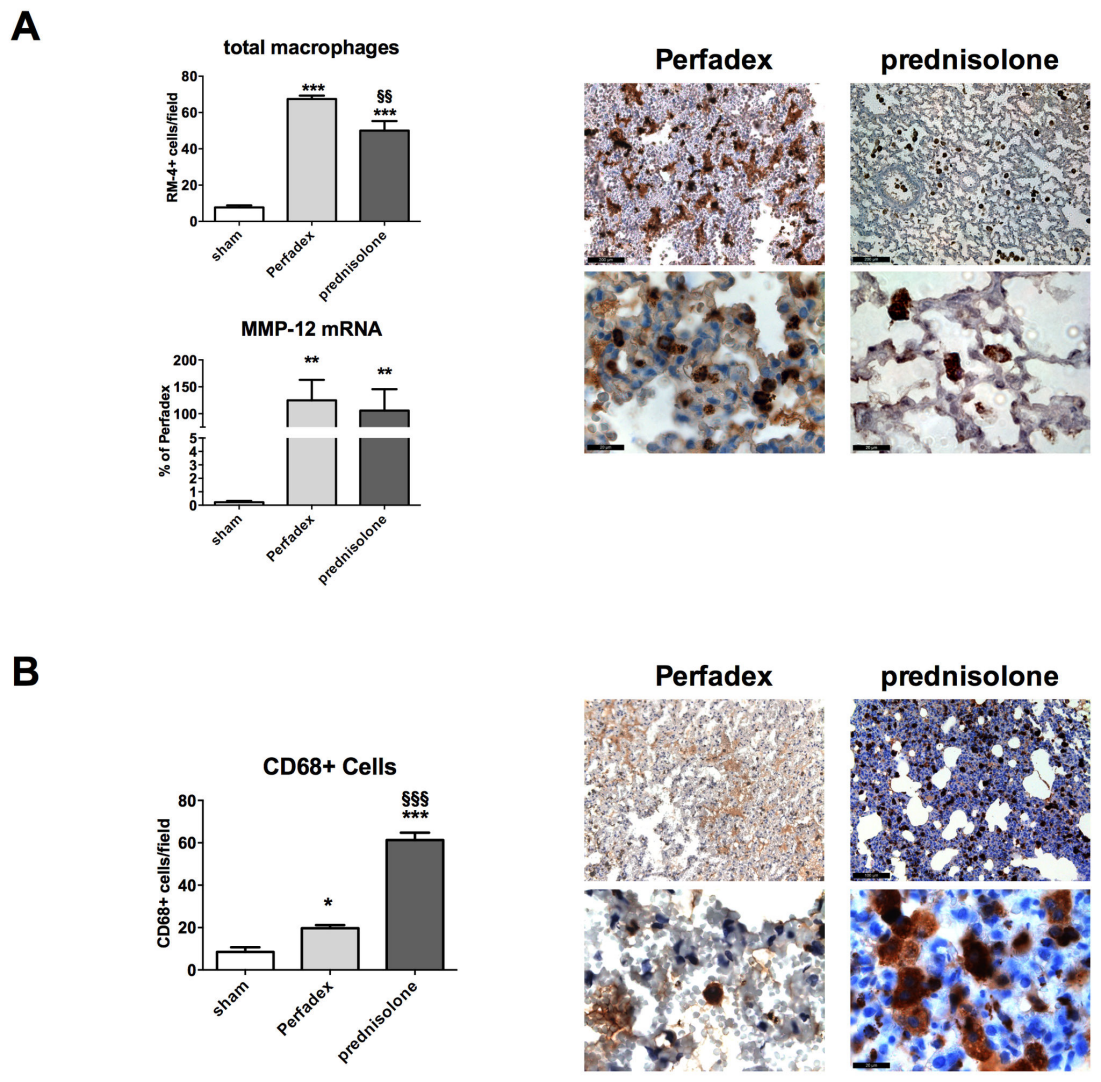
Prednisolone has no direct effect on the number of macrophages invading the grafts after LTX. (**A**) Quantification of the total number of macrophages per field. Total macrophage staining was performed by immunohistochemistry (micrographs) and macrophage counting was performed (left upper graph). The macrophage specific MMP-12 gene expression was quantified by RT-PCR (left lower graph). (**B**) Quantification of CD68 positive cells. CD68+ cells have specifically been stained for immunohistochemistry (right micrographs) and positively stained cells per field (brown) have been counted (left graph). Calibration bar for micrographs represents 100 µm (200 x magnification, upper micrographs) and 20 µm (1000 x magnification, lower micrographs). N=3 fields per slide have been evaluated. Significance level was set to P<0.05. P<0.05: *, P<0.01: **, § § and P<0.001: § § §. * = significantly different from shams and § = significantly different from Perfadex-group. RT-PCR: experiment was performed in triplicates and data are expressed as % of Perfadex group (Perfadex group has been set at 100%), protein array: pooled analysis in duplicate. ANOVA followed by Bonferroni’s post-hoc multiple comparisons test.

Prednisolone-treated animals had high gene expression levels of MMP-12 (macrophage MMP) 105.7 ± 40.0% when compared to shams 0.21 ± 0.09% (P<0.05), which corresponded to the MMP-12 expression of control animals 124.9 ± 38.0%. When directly counting the amount of macrophages in micrographs, we observed that prednisolone-treated lungs contained significantly less (50.0 ± 5.3 cells) than the corresponding controls (67.4 ± 1.9 cells; P<0.01). However, their number was significantly increased in both control and prednisolone groups when compared to shams (7.7 ± 1.1 cells; P<0.001; Figure 5A).

We observed that animals receiving prednisolone had much higher amounts of CD68+ cells/field (61.3 ± 3.5 cells) than the corresponding Perfadex-treated (19.7 ± 1.5 cells; P<0.001) or sham rats (8.5 ± 2.2 cells; P<0.001). Perfadex-treated lungs had significantly more CD68+ cells than the shams (P<0.05). The amount of CD68+ cells corresponded to the amount of total macrophages in the prednisolone group. A high number of CD68+ stained cells were found in prednisolone treated lung grafts, however their number was even higher in regions of atelectasis (Figure 5B).

### Prednisolone-pretreatment decreases ICAM-1 related pro-inflammatory cell activation

**Figure 6:**
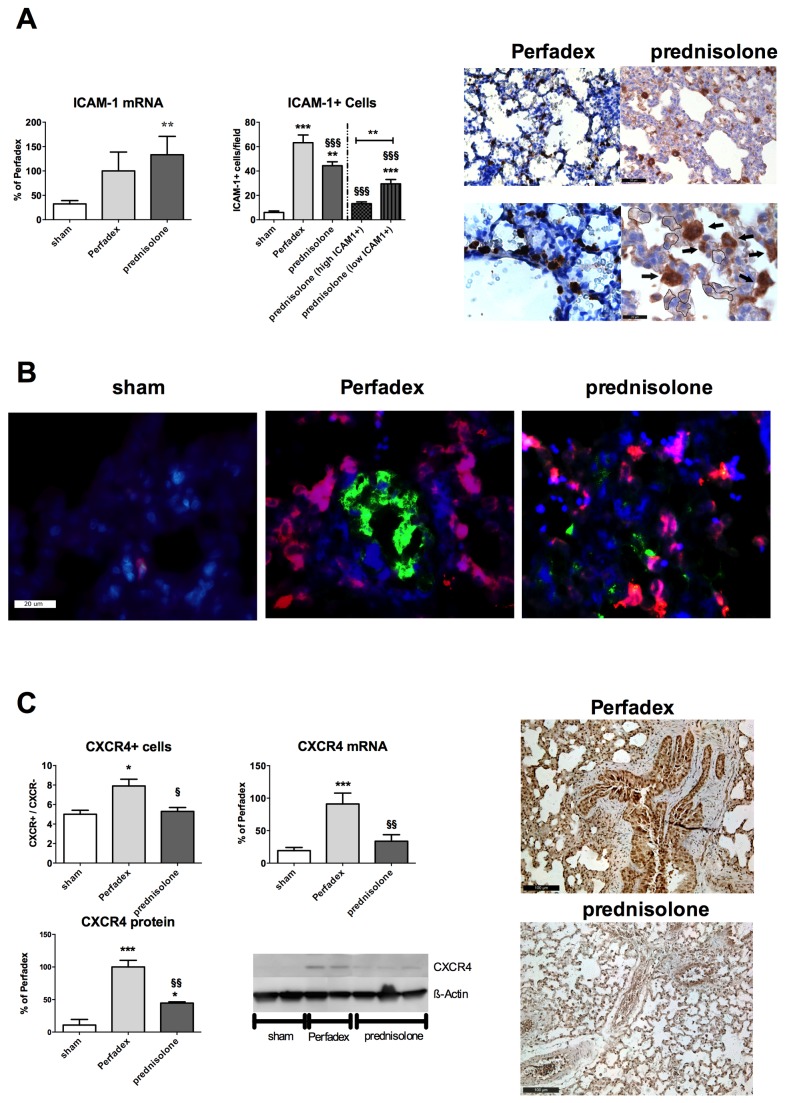
Prednisolone-pretreatment decreases ICAM-1 related pro-inflammatory cell activation. (**A**) The cellular inflammation activation marker ICAM-1 was assessed by gene expression analysis using RT-PCR (left graph) and specific ICAM-1 staining (micrographs, arrows) and counting of the ICAM-1+ cells per field (right graph). A subdivision for low ICAM-1 positive cells (micrographs, encircled areas) was performed. (**B**) Representative immunofluorescence triple staining pictures on ICAM-1 (green), macrophages (RM-4, red) and cell-cores (DAPI, blue). ICAM-1 and RM-4 co-expressing cells are colored in purple. Calibration bar represents 20 µm. (**C**) CXCR4 expression analysis was performed by immunohistochemistry (micrographs), mRNA expression analysis by RT-PCR (upper right graph) and western blot analysis (lower graph, representative western blots (right picture)). Calibration bar represents 100 µm (200 x magnification, CXCR4 stainings), 50 µm (400 x magnification, upper ICAM stainings) respectively 20 µm (1000 x magnification, lower ICAM stainings). N=3 fields per slide have been evaluated. Significance level was set to P<0.05. P<0.05: *, P<0.01: **, § § and P<0.001: § § §. * = significantly different from shams and § = significantly different from Perfadex-group. RT-PCR and western blot analysis experiments were performed in triplicates and data are expressed as % of Perfadex group (Perfadex group has been set at 100%). For CXCR4 staining, data are expressed as the ratios of CXCR4 positive vs. CXCR4 negative areas per field. ANOVA followed by Bonferroni’s post-hoc multiple comparisons test.

By measuring ICAM-1 tissue mRNA expression, the state of activation of inflammation related-cells was evaluated. We found, that only in rats receiving prednisolone perfused lungs (133.3 ± 37.6%), ICAM-1 mRNA was significantly upregulated vs. sham (32.4 ± 6.8%; P<0.01) whereas ICAM-1 mRNA was also higher in Perfadex-treated lungs (100.0 ± 38.83%) however this was not significant. In micrographs, the absolute number of ICAM-1 positive cells was significantly higher in controls (63.3 ± 6.3 cells) compared to prednisolone (44.4 ± 3.2 cells; P<0.01) or sham animals (6.1 ± 1.2 cells; P<0.001). In all animals that underwent a transplantation procedure (with or without prednisolone), ICAM-1 positive cells were significantly increased (P<0.001) compared to shams (Figure 6 A).

Immunofluorescence analysis of lung tissue revealed that prednisolone-pretreated lungs mainly contained ICAM-1 positive macrophages, whereas in Perfadex only-treated lungs, ICAM-1 was originating in large parts from other cells than macrophages (Figure 6B, red: macrophages, green: ICAM-1 positive cells, purple: ICAM-1 positive macrophages).

The gene expression of the pro-inflammatory CXCR4 was significantly upregulated in controls (91.1 ± 16.8%) when compared to sham (19.3 ± 4.9%; P<0.001) or prednisolone treated animals (33.9 ± 9.9%; P<0.01). Simultaneously, CXCR4 protein expression was significantly lower after prednisolone treatment (44.5 ± 2.0%) when compared to the corresponding controls (100.0 ± 10.2%; P<0.01). Concordantly with these observations, sham (5.00 ± 0.40 cells) and prednisolone-treated (5.29 ± 0.39 cells) lungs had significantly less CXCR4 positive cells, compared to Perfadex-treated lungs (7.96 ± 0.68 cells; P<0.05; Figure 6C).

### Prednisolone orchestrates the microenvironmental inflammatory response by inhibition of pro- and activation of anti-inflammatory pathways

**Figure 7:**
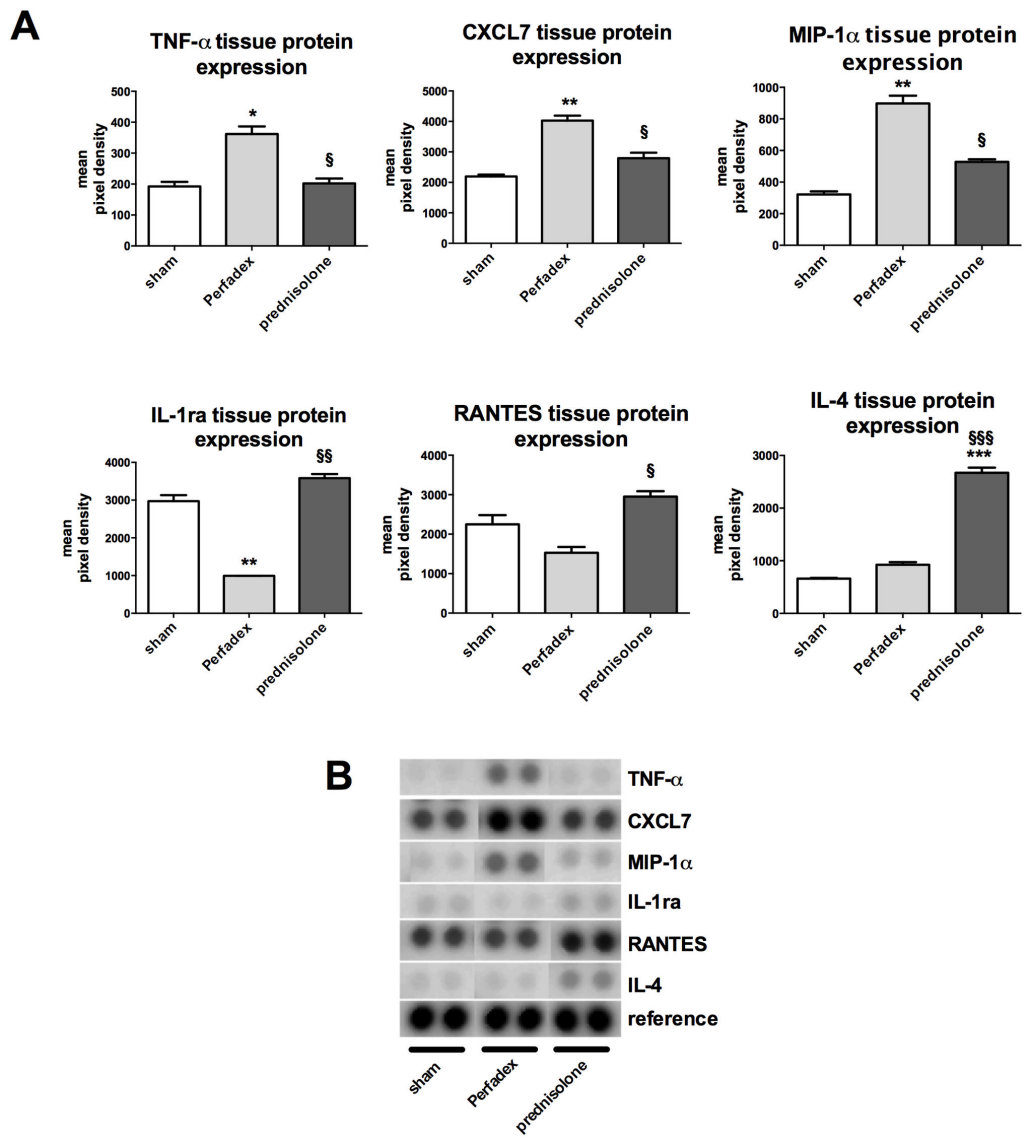
Prednisolone induces M2 macrophage polarization. (**A**) The gene expressions of the anti-inflammatory cytokine IL-13 and of a marker for M2 polarization, MRC-1, were analyzed by RT-PCR. (**B**) Selective immunohistochemical CD163 staining (brown cells) and counting of the cells (graph). N=3 fields per rat have been evaluated. Significance level was set to P<0.05. P<0.01: **, § § and P<0.001: § § §. * = significantly different from shams and § = significantly different from Perfadex-group. RT-PCR experiments were performed in triplicates and means are expressed as % of Perfadex group (Perfadex group has been set at 100%). ANOVA followed by Bonferroni’s post-hoc multiple comparisons test.

Tissue protein expression of tumor necrosis factor (TNF)-α was significantly upregulated in Perfadex-treated lungs (362.0 ± 24.42 mean pixel density) when compared to prednisolone-treated animals (202.5 ± 15.75 mean pixel density) or shams (192.2 ± 14.94 mean pixel density; P<0.05). Tissue protein expression of chemokine (C-X-C motif) ligand (CXCL) 7 was significantly upregulated in Perfadex-treated lungs (4025 ± 163.0 mean pixel density) when compared to prednisolone-treated animals (2794 ± 178.5 mean pixel density; P<0.05) or shams (2192 ± 63.88 mean pixel density; P<0.01). Tissue protein expression of macrophage inflammatory protein (MIP)-1α was significantly upregulated in Perfadex-treated lungs (898.0 ± 49.28 mean pixel density) when compared to prednisolone-treated animals (528.0 ± 16.41 mean pixel density; P<0.05) or shams (321 ± 19.35 mean pixel density; P<0.01). Tissue protein expression of interleukin-1 receptor antagonist (IL-1ra) was significantly downregulated in Perfadex-treated lungs (993.6 ± 0.44 mean pixel density) when compared to prednisolone-treated animals (3582 ± 108.4 mean pixel density; P<0.01) or shams (2972 ± 161.1 mean pixel density; P<0.01). Tissue protein expression of regulated on activation, normal T cell expressed and secreted (RANTES) was significantly downregulated in Perfadex-treated lungs (1527 ± 149.0 mean pixel density) compared to prednisolone-treated animals (2950 ± 139.0 mean pixel density; P<0.05) but not to shams (2248 ± 234.8 mean pixel density; n.s.). Finally, tissue protein expression of the anti-inflammatory IL-4 was significantly upregulated in prednisolone-treated lungs (2672 ± 96.09 mean pixel density) when compared to Perfadex-treated animals (923.1 ± 50.30 mean pixel density; P<0.001) or shams (662.1 ± 11.89 mean pixel density; P<0.001) (Figure 7A).

### Prednisolone enhances transdifferentiation of macrophages towards the anti-inflammatory M2 phenotype

Additional gene expression analysis of IL-13 revealed that its mRNA expression was significantly upregulated in prednisolone (583.8 ± 192.8%) vs. control (100.0 ± 33.5%; P<0.01) or sham animals (2.3 ± 0.4%; P<0.01).

**Figure 8:**
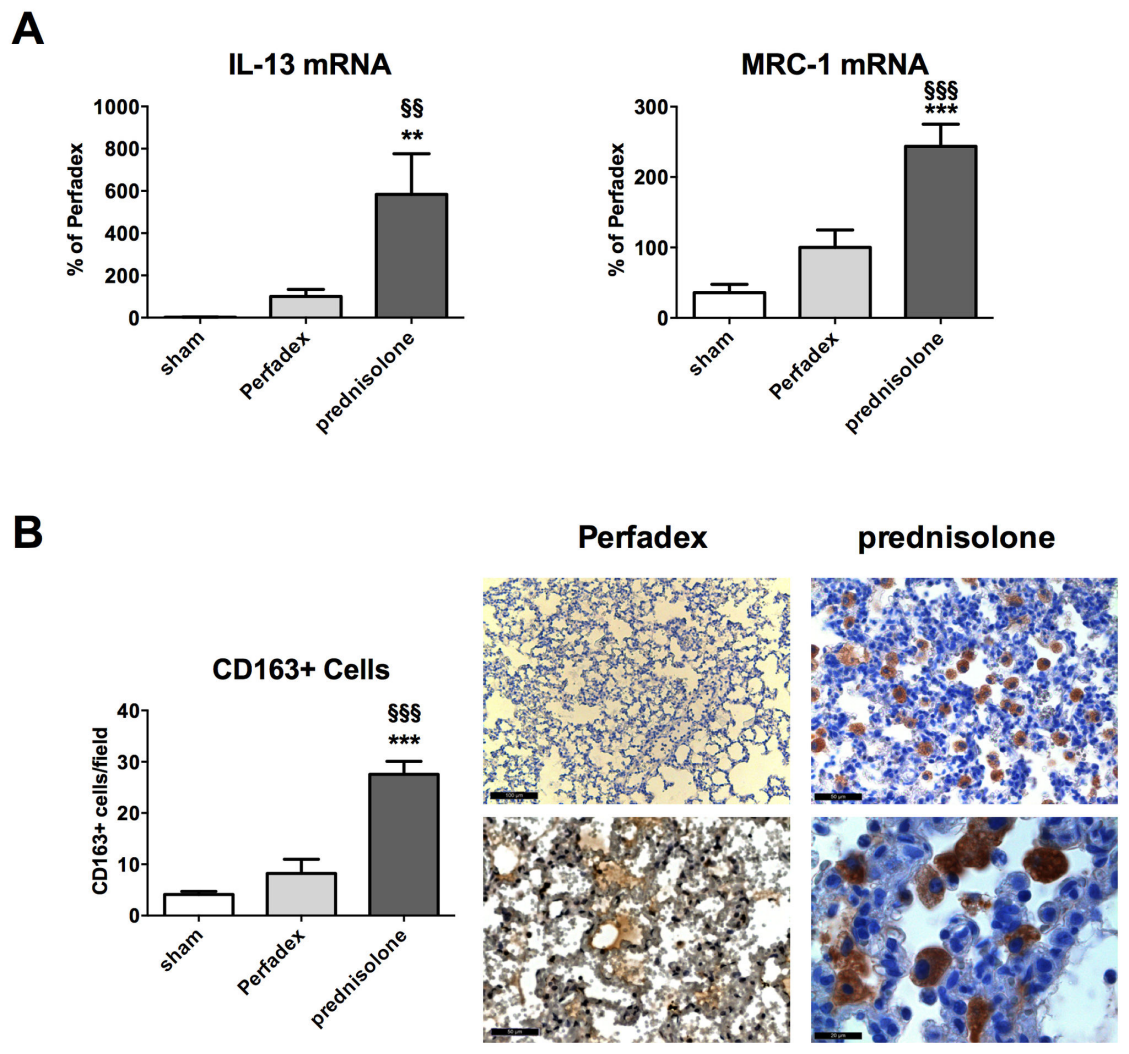
Prednisolone induces anti-inflammatory and inhibits pro-inflammatory pathways. (**A**) Tissue protein expression of TNF-α, CXCL7, MIP-1α, IL-1ra, RANTES and IL-4 were evaluated using protein arrays. Experiments using pooled samples from n=6 rats per group were performed in duplicates. (**B**) representative array pictures. Significance level was set to P<0.05. P<0.05: *, §; P<0.01: **, § § and P<0.001: § § §. * = significantly different from shams and § = significantly different from Perfadex-group Data are expressed as mean pixel densities. ANOVA followed by Bonferroni’s post-hoc multiple comparisons test.

Simultaneously the mannose receptor C type (MRC)-1 gene expression was significantly upregulated in prednisolone animals (243.6 ± 31.5%) compared to controls (100.0 ± 24.9%; P<0.001) or shams (35.8 ± 11.9%; P<0.001) indicating a M2 transdifferentiation in the prednisolone treated rats (Figure 8A).

M2 macrophages mostly express high levels of CD163. By counting CD163+ cells we observed a significantly increased number in prednisolone treated animals (27.5 ± 2.5 cells) vs. controls (8.2 ± 2.8 cells; P<0.001) or shams (4.1 ± 0.6 cells; P<0.001; Figure 8B).

## Discussion

Ischemia and reperfusion injury (IRI) represents one of the main causes of early graft dysfunction and poor outcome after transplantation [5,6,42,43]. IRI is often accompanied by tissue damage resulting in edema and with increased inflammatory cell invasion into the graft [44]. Corticoids have been used for decades to treat inflammatory disorders especially in transplantation medicine [45], where they are usually administered immediately before antigen contact or during manifest inflammation episodes i.e. during acute rejection. The approach that corticosteroids are administered via the perfusion solution to prevent damage from induced by ischemia is new and its role as additive in preservation solutions remains unclear.

In the present work we show that prednisolone, administered in combination with the perfusion solution during the phase of graft ischemia, is protective, significantly improves short-term survival and attenuates reperfusion injury in a rat model of orthotopic LTX. Animals that showed an inflammatory reaction in the reperfusion phase died of global pulmonary edema. In fact, we observed inflammation occurring in the transplanted lung also affected the right (not transplanted) lung, an observation that is not new but that has often been neglected [46]. This observation impressively shows that IRI is not only a local process but rather a systemic one.

Moreover, we could show in our orthotopic transplantation model that it makes sense to treat ischemia related effects already prophylactically at the stage of organ procurement. Our data indicates that prednisolone might interfere with hypoxia/ ischemia driven pathways such as HIF-1, a pathway that is known to strongly induce inflammation, edema (via VEGF) and organ damage [4,8,22,47]. However, the influence of prednisolone on the HIF pathway might not be strong enough, as seen in VEGF-A analysis. However the VEGF-A production is significantly blunted in prednisolone-treated lungs, it is even below values recorded in sham animals. The extent of the edema still remains higher in the prednisolone group compared to shams (Figures 2 and 3). Nevertheless, prednisolone does not only act as anti-edematous substance, its anti-inflammatory immune modulatory properties might be the most important actions of prednisolone. In an earlier work, we could already show, that protein biosynthesis is not completely blunted under cold ischemia [4]. This is important to know, as pro-inflammatory mechanisms can already be activated during the phase of conservation. On the other hand this also means that pharmacological anti-inflammatory intervention might also be effective at this stage and thus prevent deleterious effects when reperfusion commences. Inflammatory cell invasion might represent the most important point of action as neutrophils and macrophages are among the first line of attack during reperfusion and might amplify the inflammatory response and thus the graft injury (Figures 4-6). While our data clearly supports such a function, it has to be mentioned that our findings represent only a snapshot at a certain point of time. The fact that the post-inflammatory process is very dynamic can be observed in IFN-γ, IL-6 and IL-10 expression (Figure S1). These classical inflammation-modulating factors are known to play a role during almost every inflammatory process. However in our model they seem to play a minor role or might even play an opposite role to the one known from literature. IL-10 has been described as anti-inflammatory and being involved in anti-inflammatory M2 macrophage polarization [32]. However, in our model, IL-10 mRNA expression is upregulated in the Perfadex as well as in the prednisolone group and additionally its protein expression is significantly higher in the prednisolone group. The latter observation correlates with the anti-inflammatory properties of IL-10. We assume, that the mRNA upregulation of IL-10 in the Perfadex group might represent a naturally occurring counter-mechanism to limit inflammation and that this mechanism might be accelerated by prednisolone. In the present model, at least IL-4, IL-13, IL-1ra and RANTES, known molecules described to be involved in M2 polarization in previous studies, seem to have a central role in mediating the action of prednisolone into an anti-inflammatory response [26–28]. This may thus explain the reason why the amount of macrophages in the prednisolone group is similar to the Perfadex group. There might be a phenotypical difference between those two populations, indicating that the macrophages from the prednisolone group are anti-inflammatory ones. This is supported by the CD163 and MRC-1 data (Figure 8). Additionally, immunofluorescence pictures show, that in the Perfadex group the inflammation is mainly mediated via ICAM-1 positive cells, whereas this observation is missing in the prednisolone group. Whether the M2 macrophages are newly recruited or whether they derive from resident macrophages remains unclear at present and might be a subject for further investigations.

Taken together, we have shown that the application of prednisolone already in the early phase of organ procurement has a beneficial outcome. Importantly, this substance is well characterized and has an acceptable good safety profile [48,49], making it an attractive candidate for the use in human transplantations.

## Materials and Methods

### Ethics statement

The study was approved by the Animal Care and Use Committee of the state of Hessen (Regierungspräsidium Darmstadt), Germany (V54-19c20/15-F91/56). Surgery and animal care was performed in accordance with the „Guide for the care and use of laboratory animals“ (National Institutes of Health, volume 25, no. 28, revised 1996), EU Directive 86/609 EEC and German Protection of Animals Act.

### Animals

Male Sprague Dawley rats (Janvier, France) weighing 225-250g were housed in the central research facility of the Goethe-University Frankfurt. At the beginning, rats were randomized to form pairs of donor and recipient rats, which were kept together in approved plastic cages (2 animals per cage). Animals were provided with water and food *ad libitum*. Housing rooms were equipped with a 12 h light cycle.

### Lung transplantation

Lung transplantation was performed as described in detail elsewhere [4]. In short, transplantations were carried out iso-allogenically from Sprague Dawley to Sprague Dawley to avoid any kind of rejection reaction. For each of the groups surgery was performed until n=6 recipients survived. For organ preservation, 20 ml ice-cold low potassium standard dextrane containing perfusion solution Perfadex (Vitrolife, Sweden) was used to perfuse the heart lung package of the donors. Perfadex is used to quickly cool down und thus preserve lungs used for transplantation. In the *prednisolone* group 100 mg prednisolone was added to the perfusion solution. The dose of 100 mg was based on preliminary experiments. Briefly, for evaluation of the lung’s intravascular volume, organs were perfused with Perfadex substituted with Evans blue. Before perfusion, the total weight of the perfusion solution was determined. Antegrade perfusion was performed in n=4 rats by insertion of a venous catheter through the right ventricle directly into the pulmonary artery (Figure S2, A). Perfusion was carried out until Evans blue was visibly flowing out of the left atrial auricle (Figure S2, B). To verify the efficient perfusion, the lung package had to turn blue after perfusion with Evans blue (Figure S2, C). The remaining solution was weighed and the difference was calculated. Using the specific weight of Perfadex (1015,5 g/l), we were able to calculate the mean pulmonary vascular volume. As the mass oft he left lung is about 1/3 of the mass of the whole lung package, the mean left lung intravascular volume could be calculated to be 545 µl. By using 100 mg/20 ml Perfadex for perfusion, the calculated dose per rat was then 3 mg/250(g/rat) = 12 mg/kg. This dose is similar to the initiation dose used in humans to induce immune tolerance at reperfusion ranging from 6–15 mg/kg prednisolone [50–52]. The controls were perfused with Perfadex alone and did not receive prednisolone as additive. Sham animals were only subjected to a thoracotomy. Total ischemia time was set at 1h, where 45 minutes where accounted to cold ischemia and 10-15 minutes (according to the pace of the transplantation procedure) to warm ischemia. Reperfusion was only initiated after 1h total ischemia time.

Pain management was ensured with intercostal injection of 600 µl ropivacaine (0.2%) and with piritramid added to the drinking water (4.5 mg/200 ml). The recipients were observed for additional 48 h and then sacrificed under deep anesthesia. Lungs were harvested, dissected and stored at -80° or were formalin fixed (4%) and embedded in paraffin for later use [4].

### Wet-to-dry ratio

A small part of the collected lungs was minced and stored in a reaction tube and the weight determined. Then the tube was placed into an oven at 60°C for 72h. Following this, the tube was re-weighed, the weight of the tube was subtracted and the ratio between wet and dry weight was calculated [4,53].

### RNA isolation and PCR

RNA isolation and reverse transcription was performed as described elsewhere [4]. Realtime-PCR was performed using the StepOne Plus device (Applied Biosystems, USA). For rat specific primer sequences, see Table 1.

**Table 1:** Rat specific primer sequences.

	**forward**	**reverse**
CXCR4	CACCAACAGCCAGAGCGCGA	TGCGCTTCTGGTGGCCCTTG
VEGF-A	CCAGGCTGCACCCACGACAG	CGCACACCGCCATTAGGGGCA
ICAM-1	CGCAGTCCTCGGCTTCTGCC	CGCAGTCCTCGGCTTCTGCC
β-Actin	CTTGCAGCTCCTCCGTCGCC	CTTGCTCTGGGCCTCGTCGC
CD 163	TGGGATCGCCGTGACGCTTC	CAGCGACTGCCTCCACCGAC
IL-4	GGCTTCCAGGGTGCTTCGCAA	GTGGACTCATTCACGGTGCAGC
IL-13	GTGGTCTTGCCACCCCAGGG	CGCCAGCTGTCAGGTCCACG
MRC-1	ACGGGTGACCCTTCGGGTGA	GCCGCAACACCGGCACCTAT
MMP-2	CAAGTTCCCCGGCGATGTC	TTCTGGTCAAGGTCACCTGTC
MMP-12	TTGGCCATTCCTTGGGGCTGC	TGTTGGTGGCTGGACTCCCAGG

### Protein isolation, Western Blot, ELISA and protein arrays

Proteins were isolated according to a standard protocol [4,54,55]. 10 % and 7.5 % SDS-gels were loaded with 50 µg protein. Proteins were detected on Hybond C supermembrane (Amersham Pharmacia Biotech, UK) with Spectra brood range marker (Fermentas, Germany) as a standard. The blots were probed with antibodies against: CXCR4 (Abcam, UK), VEGF (Abcam, UK) and β-Actin (Abcam, UK). Digitalization and evaluation of the blots was performed with a Kodak Imager (Carestream, Germany). For serum sampling, blood was withdrawn and clotting was performed for 15 minutes followed by centrifugation at 3.000 RPM for 10 minutes at 4°C. Serum supernatant was used for ELISA on rat carbonic anhydrase IX (Hölzel Diagnostika, Germany), rat VEGF-A (R&D Systems, Germany), rat IFNγ and rat IL-6 (Thermo, Fisher, Germany) which were performed according to the manufacturer’s protocol. For rat cytokine array (Proteome Profiler, R&D systems, Germany) whole lung protein lysates were pooled from each group in equal amounts to obtain 300 µg protein solution for each group. Membranes were then incubated and developed according to the manufacturer’s protocol. Detection of the luminescence was performed with Kodak-Imager (Carestream, Germany).

### Histology immunohistochemistry and immunofluorescence

Preparation of paraffin embedded samples was performed as described elsewhere [4]. Samples were stained with H&E (AppliChem, Germany) according to the manufacturer’s protocol. Immunohistochemistry was performed by using CD 163 (AbD Serotec, Germany), CD 68 (Millipore, Germany), ICAM-1 (Labomics, Belgium), RM-4 pan-macrophage/dendritic cell marker (TransGenic Inc., Kobe, Japan) and CXCR4 (Abcam, UK) antibodies [4]. For detection, corresponding secondary antibodies labelled with biotin were used prior to detection with streptavidin-HRP (AXXORA, Germany). Development was performed with DAB and counterstaining was done with haematoxylin. For immunofluorescence we used mouse monoclonal anti rat macrophage/dendritic cells RM4 (TransGenic Inc., Kobe, Japan) and the rabbit polyclonal anti CD54/ICAM-1 (Biozol Diagnostica, Germany). Detection was performed by incubating the slides with secondary anti-mouse PE (eBioscience, USA) conjugated and anti rabbit Alexa Fluor^®^ 488 (Molecular Biotechnology, Germany) conjugated antibodies. Core staining was carried out with DAPI (Vector Laboratories, USA) staining. Pictures were taken for every fluorescent dye and images were merged to show co-expression of ICAM-1 and RM4.

Histological examinations were conducted in a blinded fashion. Three slides per animal were stained. Pictures were then taken from the same lung area (peripheral longitudinal section) and 3 fields per slide were photographed randomly. Evaluation of the edema was performed by analyzing the amount of tissue (colored area) vs. no tissue (uncolored area) per field. The algorithm calculated the amount of pixel that were covered by the stained tissue in the respective pictures and calculated the amount of pixel of the uncolored area and expressed the values as % of the total pixel. We define colored pixel as being a part of tissue, concluding that all stained areas are covered with tissue. This technique was already validated elsewhere and showed high accuracy [4]. Images were taken with the Leica DM5000B microscope, stored in tagged image file format and analyzed using an automatized Matlab script (The Mathworks, USA) programmed by the authors. The routine determines the color-values of pixels in the red-green-blue space (RGB), thus measuring the relative area occupied by blue, red and green staining per image. Quantification was done by setting colored pixels in proportion to the total number of all pixels and the number of uncolored pixels. As internal negative control, isotype IgG stainings were performed (pictures not shown).

### Statistical analysis

Statistical analysis was performed with GraphPad Prism® 5.02 software (GraphPad Software, Inc., California, USA). Results are expressed as means ± standard error of the mean (SEM). Statistical significance was calculated using ANOVA followed by a post hoc analysis using Bonferroni’s multiple comparison tests. Statistical significance was set to p< 0.05 (* p<0.05, ** p<0.01, *** p<0.001).

## Supporting Information

Figure S1 **Expression of IFN-γ, IL-6 and IL-10 following LTX.** (**A**) Circulating levels of IFN-γ were detected using ELISA (left graph). Tissue protein expression of IFN-γ was measured using a protein array middle graph. Exemplary pictures of the profiler for IFN-γ are shown on the right. (**B**) Circulating IL-6 levels were detected using ELISA (left graph). Tissue protein expression of IL-6 was measured using a protein array middle graph. Exemplary pictures of the profiler for IL-6 are shown on the right. (**C**) IL-10 gene expression was assessed by RT-PCR analysis (left graph). Tissue protein expression of IL-10 was measured using a protein array middle graph. Exemplary pictures of the profiler for IL-6 are shown on the right. Significance level was set to P<0.05. P<0.05: *, §, P<0.01: ** and P<0.001: ***. * = significantly different from shams and § = significantly different from Perfadex-group. RT-PCR experiments were performed in triplicates and data are expressed as % of Perfadex group (Perfadex group has been set at 100%), protein array: pooled analysis in duplicate. ANOVA followed by Bonferroni’s post-hoc multiple comparisons test.
(TIFF)

Figure S2 **Prednisolone dose finding pilot experiment.** (**A**) Insertion of the perfusion catheter through an incision in the right ventricle directly into the pulmonary artery (*). (**B**) Begin of the perfusion procedure with Perfadex substituted with Evans blue (n=4 rats). Perfusion was stopped at the moment where the blue color was flowing out of the left atrial auricle (arrow). (**C**) At the end of the perfusion, the lungs turned completely blue as proof of efficient perfusion. The remaining volume of perfusion solution was determined and the difference was calculated. After determination of the left and right lung weights, the corresponding final left and right lung volumes could be extrapolated.
(TIFF)

## References

[1] Wohlschläger J, Sommerwerck U, Jonigk D, Rische J, Baba HA et al. (2011) [Lung transplantation and rejection. Basic principles, clinical aspects and histomorphology]. Pathologe 32: 104-112. doi:10.1007/s00292-010-1403-1. PubMed: 21424408. 21424408 10.1007/s00292-010-1403-1

[2] Boucek MM, Aurora P, Edwards LB, Taylor DO, Trulock EP et al. (2007) Registry of the International Society for Heart and Lung Transplantation: tenth official pediatric heart transplantation report--2007. J Heart Lung Transplant 26: 796-807. doi:10.1016/j.healun.2007.06.006. PubMed: 17692783. 17692783 10.1016/j.healun.2007.06.006

[3] Trulock EP, Christie JD, Edwards LB, Boucek MM, Aurora P et al. (2007) Registry of the International Society for Heart and Lung Transplantation: twenty-fourth official adult lung and heart-lung transplantation report-2007. J Heart Lung Transplant 26: 782-795. doi:10.1016/j.healun.2007.06.003. PubMed: 17692782. 17692782 10.1016/j.healun.2007.06.003

[4] Paulus P, Ockelmann P, Tacke S, Karnowski N, Ellinghaus P et al. (2012) Deguelin attenuates reperfusion injury and improves outcome after orthotopic lung transplantation in the rat. PLOS ONE 7: e39265. doi:10.1371/journal.pone.0039265. PubMed: 22745725. 22745725 10.1371/journal.pone.0039265 PMC3380011

[5] Lee JC, Christie JD (2011) Primary graft dysfunction. Clin Chest Med 32: 279-293. doi:10.1016/j.ccm.2011.02.007. PubMed: 21511090. 21511090 10.1016/j.ccm.2011.02.007

[6] Aharinejad S, Schäfer R, Krenn K, Zuckermann A, Schneider B et al. (2007) Donor myocardial HIF-1alpha is an independent predictor of cardiac allograft dysfunction: a 7-year prospective, exploratory study. Am J Transplant 7: 2012-2019. doi:10.1111/j.1600-6143.2007.01875.x. PubMed: 17617866. 17617866 10.1111/j.1600-6143.2007.01875.x

[7] Krüger B, Krick S, Dhillon N, Lerner SM, Ames S et al. (2009) Donor Toll-like receptor 4 contributes to ischemia and reperfusion injury following human kidney transplantation. Proc Natl Acad Sci U S A 106: 3390-3395. doi:10.1073/pnas.0810169106. PubMed: 19218437. 19218437 10.1073/pnas.0810169106 PMC2651292

[8] Abraham D, Taghavi S, Riml P, Paulus P, Hofmann M et al. (2002) VEGF-A and -C but not -B mediate increased vascular permeability in preserved lung grafts. Transplantation 73: 1703-1706. doi:10.1097/00007890-200206150-00003. PubMed: 12084990. 12084990 10.1097/00007890-200206150-00003

[9] Paulus P, Jennewein C, Zacharowski K (2011) Biomarkers of endothelial dysfunction: can they help us deciphering systemic inflammation and sepsis? Biomarkers 16 Suppl 1: S11-S21. doi:10.3109/1354750X.2011.587893. PubMed: 21707440. 21707440 10.3109/1354750X.2011.587893

[10] Wissink S, van Heerde EC, vand der Burg B, van der Saag PT (1998) A dual mechanism mediates repression of NF-kappaB activity by glucocorticoids. Molecular endocrinology 12: 355-363. 9514153 10.1210/mend.12.3.0081

[11] Thompson EB, Lippman ME (1974) Mechanism of action of glucocorticoids. Metab Clin Exp 23: 159-202. doi:10.1016/0026-0495(74)90113-9. PubMed: 4359315. 4359315 10.1016/0026-0495(74)90113-9

[12] Parrillo JE, Fauci AS (1979) Mechanisms of glucocorticoid action on immune processes. Annu Rev Pharmacol Toxicol 19: 179-201. doi:10.1146/annurev.pa.19.040179.001143. PubMed: 222199. 222199 10.1146/annurev.pa.19.040179.001143

[13] de Jong EC, Vieira PL, Kalinski P, Kapsenberg ML (1999) Corticosteroids inhibit the production of inflammatory mediators in immature monocyte-derived DC and induce the development of tolerogenic DC3. J Leukoc Biol 66: 201-204. PubMed: 10449154. 10449154 10.1002/jlb.66.2.201

[14] Barbulescu K, Becker C, Meyer zum Büschenfelde KH, Neurath MF (1998) Regulation of protein-DNA interactions at the interferon-gamma gene promoter by corticosteroids. Ann N Y Acad Sci 859: 319-322. doi:10.1111/j.1749-6632.1998.tb11155.x. PubMed: 9928413. 9928413 10.1111/j.1749-6632.1998.tb11155.x

[15] Felszeghy K, Banisadr G, Rostène W, Nyakas C, Haour F (2004) Dexamethasone downregulates chemokine receptor CXCR4 and exerts neuroprotection against hypoxia/ischemia-induced brain injury in neonatal rats. Neuroimmunomodulation 11: 404-413. doi:10.1159/000080151. PubMed: 15467356. 15467356 10.1159/000080151

[16] Blotta MH, DeKruyff RH, Umetsu DT (1997) Corticosteroids inhibit IL-12 production in human monocytes and enhance their capacity to induce IL-4 synthesis in CD4+ lymphocytes. J Immunol 158: 5589-5595. PubMed: 9190905. 9190905

[17] Chung KF, Adcock IM (2005) Signalling and transcriptional regulation in inflammatory and immune cells: importance in lung biology and disease. Eur Respir J 26: 762-763. doi:10.1183/09031936.05.00093305. PubMed: 16264033. 16264033 10.1183/09031936.05.00093305

[18] Zhao M, Fernandez LG, Doctor A, Sharma AK, Zarbock A et al. (2006) Alveolar macrophage activation is a key initiation signal for acute lung ischemia-reperfusion injury. Am J Physiol Lung Cell Mol Physiol 291: L1018-L1026. doi:10.1152/ajplung.00086.2006. PubMed: 16861385. 16861385 10.1152/ajplung.00086.2006

[19] Fiser SM, Tribble CG, Long SM, Kaza AK, Kern JA et al. (2001) Pulmonary macrophages are involved in reperfusion injury after lung transplantation. Ann Thorac Surg 71: 1134-1138; discussion 1138-1139 doi:10.1016/S0003-4975(01)02407-9. PubMed: 11308149. 11308149 10.1016/s0003-4975(01)02407-9

[20] Ross SD, Tribble CG, Gaughen JR Jr., Shockey KS, Parrino PE et al. (1999) Reduced neutrophil infiltration protects against lung reperfusion injury after transplantation. Ann Thorac Surg 67: 1428-1433; discussion 1434 doi:10.1016/S0003-4975(99)00248-9. PubMed: 10355425. 10355425 10.1016/s0003-4975(99)00248-9

[21] Chao J, Wood JG, Gonzalez NC (2009) Alveolar hypoxia, alveolar macrophages, and systemic inflammation. Respir Res 10: 54. doi:10.1186/1465-9921-10-54. PubMed: 19545431. 19545431 10.1186/1465-9921-10-54 PMC2705912

[22] Eltzschig HK, Carmeliet P (2011) Hypoxia and inflammation. N Engl J Med 364: 656-665. doi:10.1056/NEJMra0910283. PubMed: 21323543. 21323543 10.1056/NEJMra0910283 PMC3930928

[23] Moldobaeva A, van Rooijen N, Wagner EM (2011) Effects of ischemia on lung macrophages. PLOS ONE 6: e26716. doi:10.1371/journal.pone.0026716. PubMed: 22110592. 22110592 10.1371/journal.pone.0026716 PMC3217923

[24] Beck-Schimmer B, Schwendener R, Pasch T, Reyes L, Booy C et al. (2005) Alveolar macrophages regulate neutrophil recruitment in endotoxin-induced lung injury. Respir Res 6: 61. doi:10.1186/1465-9921-6-61. PubMed: 15972102. 15972102 10.1186/1465-9921-6-61 PMC1188075

[25] Murray PJ, Wynn TA (2011) Protective and pathogenic functions of macrophage subsets. Nat Rev Immunol 11: 723-737. doi:10.1038/nri3073. PubMed: 21997792. 21997792 10.1038/nri3073 PMC3422549

[26] Pinto AR, Paolicelli R, Salimova E, Gospocic J, Slonimsky E et al. (2012) An abundant tissue macrophage population in the adult murine heart with a distinct alternatively-activated macrophage profile. PLOS ONE 7: e36814. doi:10.1371/journal.pone.0036814. PubMed: 22590615. 22590615 10.1371/journal.pone.0036814 PMC3349649

[27] Martinez FO, Helming L, Gordon S (2009) Alternative activation of macrophages: an immunologic functional perspective. Annu Rev Immunol 27: 451-483. doi:10.1146/annurev.immunol.021908.132532. PubMed: 19105661. 19105661 10.1146/annurev.immunol.021908.132532

[28] Pelegrin P, Surprenant A (2009) Dynamics of macrophage polarization reveal new mechanism to inhibit IL-1beta release through pyrophosphates. EMBO J 28: 2114-2127. doi:10.1038/emboj.2009.163. PubMed: 19536133. 19536133 10.1038/emboj.2009.163 PMC2699392

[29] Lim HY, Müller N, Herold MJ, van den Brandt J, Reichardt HM (2007) Glucocorticoids exert opposing effects on macrophage function dependent on their concentration. Immunology 122: 47-53. doi:10.1111/j.1365-2567.2007.02611.x. PubMed: 17451463. 17451463 10.1111/j.1365-2567.2007.02611.x PMC2265978

[30] Ehrchen J, Steinmüller L, Barczyk K, Tenbrock K, Nacken W et al. (2007) Glucocorticoids induce differentiation of a specifically activated, anti-inflammatory subtype of human monocytes. Blood 109: 1265-1274. PubMed: 17018861. 17018861 10.1182/blood-2006-02-001115

[31] Hebenstreit D, Wirnsberger G, Horejs-Hoeck J, Duschl A (2006) Signaling mechanisms, interaction partners, and target genes of STAT6. Cytokine Growth Factor Rev 17: 173-188. doi:10.1016/j.cytogfr.2006.01.004. PubMed: 16540365. 16540365 10.1016/j.cytogfr.2006.01.004

[32] Biswas SK, Mantovani A (2010) Macrophage plasticity and interaction with lymphocyte subsets: cancer as a paradigm. Nat Immunol 11: 889-896. doi:10.1038/ni.1937. PubMed: 20856220. 20856220 10.1038/ni.1937

[33] Mocellin S, Panelli MC, Wang E, Nagorsen D, Marincola FM (2003) The dual role of IL-10. Trends Immunol 24: 36-43. doi:10.1016/S1471-4906(02)00009-1. PubMed: 12495723. 12495723 10.1016/s1471-4906(02)00009-1

[34] Sun L, Cornell TT, LeVine A, Berlin AA, Hinkovska-Galcheva V et al. (2013) Dual role of interleukin-10 in the regulation of respiratory syncitial virus (RSV)-induced lung inflammation. Clin Exp Immunol 172: 263-279. doi:10.1111/cei.12059. PubMed: 23574323. 23574323 10.1111/cei.12059 PMC3628329

[35] Sica A, Mantovani A (2012) Macrophage plasticity and polarization: in vivo veritas. J Clin Invest 122: 787-795. doi:10.1172/JCI59643. PubMed: 22378047. 22378047 10.1172/JCI59643 PMC3287223

[36] Gordon S, Martinez FO (2010) Alternative activation of macrophages: mechanism and functions. Immunity 32: 593-604. doi:10.1016/j.immuni.2010.05.007. PubMed: 20510870. 20510870 10.1016/j.immuni.2010.05.007

[37] Gordon S (2003) Alternative activation of macrophages. Nat Rev Immunol 3: 23-35. doi:10.1038/nri978. PubMed: 12511873. 12511873 10.1038/nri978

[38] Iwata T, Chiyo M, Yoshida S, Smith GN Jr., Mickler EA et al. (2008) Lung transplant ischemia reperfusion injury: metalloprotease inhibition down-regulates exposure of type V collagen, growth-related oncogene-induced neutrophil chemotaxis, and tumor necrosis factor-alpha expression. Transplantation 85: 417-426. PubMed: 18322435. 18322435 10.1097/TP.0b013e31815e91b6

[39] Song J, Wu C, Zhang X, Sorokin LM (2013) In vivo processing of CXCL5 (LIX) by matrix metalloproteinase (MMP)-2 and MMP-9 promotes early neutrophil recruitment in IL-1beta-induced peritonitis. J Immunol 190: 401-410. doi:10.4049/jimmunol.1202286. PubMed: 23225890. 23225890 10.4049/jimmunol.1202286

[40] Corbel M, Belleguic C, Boichot E, Lagente V (2002) Involvement of gelatinases (MMP-2 and MMP-9) in the development of airway inflammation and pulmonary fibrosis. Cell Biol Toxicol 18: 51-61. doi:10.1023/A:1014471213371. PubMed: 11991086. 11991086 10.1023/a:1014471213371

[41] Belvisi MG (2004) Regulation of inflammatory cell function by corticosteroids. Proc Am Thorac Soc 1: 207-214. doi:10.1513/pats.200402-002MS. PubMed: 16113436. 16113436 10.1513/pats.200402-002MS

[42] Linfert D, Chowdhry T, Rabb H (2009) Lymphocytes and ischemia-reperfusion injury. Transplant Rev (Orlando) 23: 1-10. doi:10.1016/S0955-470X(08)00098-0. PubMed: 19027612. 19027612 10.1016/j.trre.2008.08.003 PMC2651229

[43] Peeters P, Vanholder R (2008) Therapeutic interventions favorably influencing delayed and slow graft function in kidney transplantation: mission impossible? Transplantation 85: S31-S37. doi:10.1097/TP.0b013e318169c548. PubMed: 18401261. 18401261 10.1097/TP.0b013e318169c548

[44] Ng CS, Wan S, Arifi AA, Yim AP (2006) Inflammatory response to pulmonary ischemia-reperfusion injury. Surg Today 36: 205-214. doi:10.1007/s00595-005-3124-2. PubMed: 16493527. 16493527 10.1007/s00595-005-3124-2

[45] Rogatsky I, Ivashkiv LB (2006) Glucocorticoid modulation of cytokine signaling. Tissue Antigens 68: 1-12. doi:10.1111/j.1399-0039.2006.00599.x. PubMed: 16774534. 16774534 10.1111/j.1399-0039.2006.00599.x

[46] McAdams HP, Erasmus JJ, Palmer SM (2001) Complications (excluding hyperinflation) involving the native lung after single-lung transplantation: incidence, radiologic features, and clinical importance. Radiology 218: 233-241. PubMed: 11152808. 11152808 10.1148/radiology.218.1.r01ja45233

[47] Eltzschig HK (2011) Targeting Hypoxia-induced Inflammation. Anesthesiology 114: 239-242. doi:10.1097/ALN.0b013e3182070c66. PubMed: 21239967. 21239967 10.1097/ALN.0b013e3182070c66 PMC3074366

[48] Ferrero V, Ribichini F, Rognoni A, Marino P, Brunelleschi S et al. (2007) Comparison of efficacy and safety of lower-dose to higher-dose oral prednisone after percutaneous coronary interventions (the IMPRESS-LD study). Am J Cardiol 99: 1082-1086. doi:10.1016/j.amjcard.2006.11.064. PubMed: 17437731. 17437731 10.1016/j.amjcard.2006.11.064

[49] Novick RJ, Menkis AH, McKenzie FN, Reid KR, Pflugfelder PW et al. (1991) The safety of low-dose prednisone before and immediately after heart-lung transplantation. Ann Thorac Surg 51: 642-645. doi:10.1016/0003-4975(91)90325-K. PubMed: 2012424. 2012424 10.1016/0003-4975(91)90325-k

[50] Bhorade SM, Stern E (2009) Immunosuppression for lung transplantation. Proc Am Thorac Soc 6: 47-53. doi:10.1513/pats.200808-096GO. PubMed: 19131530. 19131530 10.1513/pats.200808-096GO

[51] Follette DM, Rudich SM, Babcock WD (1998) Improved oxygenation and increased lung donor recovery with high-dose steroid administration after brain death. J Heart Lung Transplant 17: 423-429. PubMed: 9588588. 9588588

[52] Sleiman C, Mal H, Fournier M, Duchatelle JP, Icard P et al. (1995) Pulmonary reimplantation response in single-lung transplantation. Eur Respir J 8: 5-9. doi:10.1183/09031936.95.08010005. PubMed: 7744193. 7744193 10.1183/09031936.95.08010005

[53] Abraham D, Krenn K, Seebacher G, Paulus P, Klepetko W et al. (2004) Upregulated hypoxia-inducible factor-1 DNA binding activity to the vascular endothelial growth factor-A promoter mediates increased vascular permeability in donor lung grafts. Ann Thorac Surg 77: 1751-1755. doi:10.1016/j.athoracsur.2003.10.050. PubMed: 15111179. 15111179 10.1016/j.athoracsur.2003.10.050

[54] Krenn K, Klepetko W, Taghavi S, Paulus P, Aharinejad S (2007) Vascular endothelial growth factor increases pulmonary vascular permeability in cystic fibrosis patients undergoing lung transplantation. Eur J Cardiothorac Surg 32: 35-41. doi:10.1016/j.ejcts.2007.04.006. PubMed: 17481912. 17481912 10.1016/j.ejcts.2007.04.006

[55] Paulus P, Stanley ER, Schäfer R, Abraham D, Aharinejad S (2006) Colony-stimulating factor-1 antibody reverses chemoresistance in human MCF-7 breast cancer xenografts. Cancer Res 66: 4349-4356. doi:10.1158/0008-5472.CAN-05-3523. PubMed: 16618760. 16618760 10.1158/0008-5472.CAN-05-3523

