# Supplementary figures and images for: Prednisolone as Preservation Additive Prevents from Ischemia Reperfusion Injury in a Rat Model of Orthotopic Lung Transplantation
Source: PLoS One. 2013 Aug 29;8(8):e73298. doi: 10.1371/journal.pone.0073298 (PMC3756949; doi:10.1371/journal.pone.0073298)

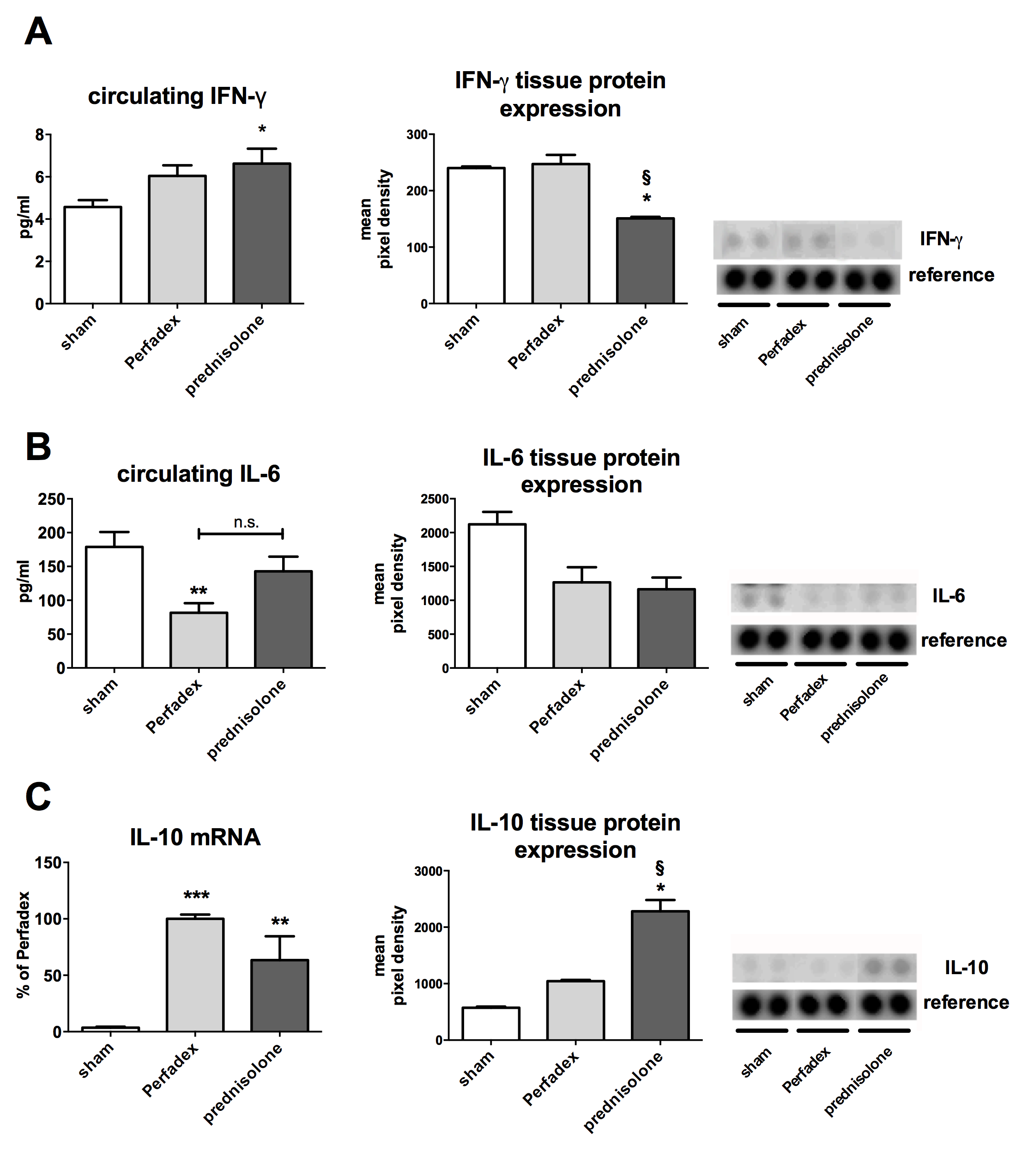

Supplement: Figure S1 — Expression of IFN-γ, IL-6 and IL-10 following LTX. (A) Circulating levels of IFN-γ were detected using ELISA (left graph). Tissue protein expression of IFN-γ was measured using a protein array middle graph. Exemplary pictures of the profiler for IFN-γ are shown on the right. (B) Circulating IL-6 levels were detected using ELISA (left graph). Tissue protein expression of IL-6 was measured using a protein array middle graph. Exemplary pictures of the profiler for IL-6 are shown on the right. (C) IL-10 gene expression was assessed by RT-PCR analysis (left graph). Tissue protein expression of IL-10 was measured using a protein array middle graph. Exemplary pictures of the profiler for IL-6 are shown on the right. Significance level was set to P<0.05. P<0.05: *, §, P<0.01: ** and P<0.001: ***. * = significantly different from shams and § = significantly different from Perfadex-group. RT-PCR experiments were performed in triplicates and data are expressed as % of Perfadex group (Perfadex group has been set at 100%), protein array: pooled analysis in duplicate. ANOVA followed by Bonferroni’s post-hoc multiple comparisons test. (TIFF) [file pone.0073298.s001.tiff]

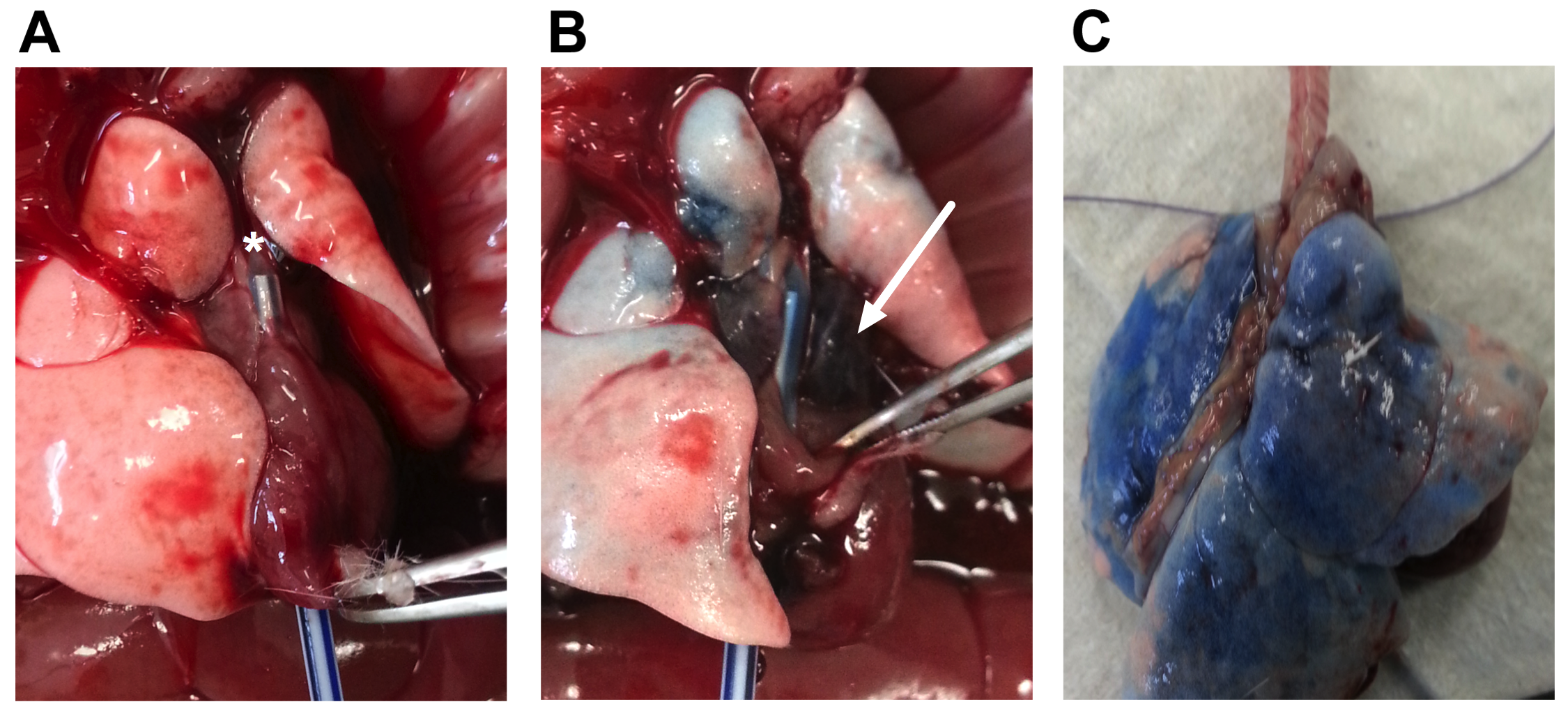

Supplement: Figure S2 — Prednisolone dose finding pilot experiment. (A) Insertion of the perfusion catheter through an incision in the right ventricle directly into the pulmonary artery (*). (B) Begin of the perfusion procedure with Perfadex substituted with Evans blue (n=4 rats). Perfusion was stopped at the moment where the blue color was flowing out of the left atrial auricle (arrow). (C) At the end of the perfusion, the lungs turned completely blue as proof of efficient perfusion. The remaining volume of perfusion solution was determined and the difference was calculated. After determination of the left and right lung weights, the corresponding final left and right lung volumes could be extrapolated. (TIFF) [file pone.0073298.s002.tiff]
